# Accurate Range Modeling for High-Resolution Spaceborne Synthetic Aperture Radar

**DOI:** 10.3390/s24103119

**Published:** 2024-05-14

**Authors:** Haisheng Li, Junshe An, Xiujie Jiang

**Affiliations:** 1 Key Laboratory of Electronics and Information Technology for Space Systems, National Space Science Center, Chinese Academy of Sciences, Beijing 100190, China; 2 University of Chinese Academy of Sciences, Beijing 100049, China

**Keywords:** synthetic aperture radar (SAR), range model, high resolution, nonlinear trajectory, space geometry

## Abstract

Spaceborne synthetic aperture radar (SAR) is an advanced microwave imaging technology that provides all-weather and all-day target information. However, as spaceborne SAR resolution improves, traditional echo signal models based on airborne SAR design become inadequate due to the curved orbit, Earth rotation, and increased propagation distance. In this study, we propose an accurate range model for high-resolution spaceborne SAR by analyzing motion trajectory and Doppler parameters from the perspective of the space geometry of spaceborne SAR. We evaluate the accuracy of existing range models and propose an advanced equivalent squint range model (AESRM) that accurately fits the actual range history and compensates for high-order term errors by introducing third-order and fourth-order error terms while maintaining the simplicity of the traditional model. The proposed AESRM’s concise two-dimensional frequency spectrum form facilitates the design of imaging algorithms. Point target simulations confirm the effectiveness of the proposed AESRM, demonstrating significant improvements in fitting accuracy for range histories characterized by nonlinear trajectories. The developed AESRM provides a robust foundation for designing imaging algorithms and enables higher resolution and more accurate radar imaging.

## 1. Introduction

As a representative high-resolution microwave imaging radar, spaceborne synthetic aperture radar (SAR) integrates pulse compression and synthetic aperture techniques to acquire two-dimensional high-resolution radar images [1,2]. It provides rich target information in all-weather and all-day conditions and has been widely applied in civilian and military fields, making it a focus of current research in high-resolution Earth observation technology [3,4]. The continuous development of spaceborne SAR technology has led to the spatial resolution being continuously improved from the tens of meters level to the meter and sub-meter levels [5,6,7,8]. However, when the resolution is improved to the sub-meter level, many assumptions and approximations in traditional imaging algorithms will no longer hold due to the high speed of spaceborne SAR, curved orbit, long propagation distance, and Earth rotation, which pose significant challenges to the signal processing of high-resolution spaceborne SAR [9,10,11,12,13].

The echo signal model serves as the foundation for frequency domain imaging algorithm design and determines the difficulty and accuracy of algorithm development [6,14,15,16]. Traditional echo signal models are based on airborne SAR design, assuming the stop-and-go approximation and straight-line trajectory. However, as spaceborne SAR resolution improves, the approximations in traditional signal models introduce phase errors, which affect the focusing effect [17]. The stop-and-go approximation neglects the motion of the radar platform, leading to the generation of fast-time and slow-time effects [12]. To address the errors caused by the stop-and-go approximation, precise echo models under non-stop-and-go conditions can be established [18], or existing algorithms can be analyzed and improved [9,12].

With the demand for higher resolution, the nonlinear motion trajectory of spaceborne SAR becomes more pronounced. The conventional hyperbolic range Equation (CHRE) is based on the assumption of a straight-line trajectory, which approximates the orbital motion as a straight line for low-resolution spaceborne SAR systems using equivalent velocity and squint angle [14]. Even for spaceborne SAR operating in broadside mode, there is still a certain squint angle due to factors such as Earth curvature and rotation. Therefore, a model known as the equivalent squint range model (ESRM), which essentially remains a hyperbolic equation, is commonly employed. However, as resolution increases, the synthetic aperture time and the curved orbit introduce a slant range error, which affects azimuth focusing. The CHRE and ESRM calculations solely rely on the Doppler centroid and Doppler frequency modulation (FM) rate and can accurately represent the real range history only up to the second-order term [4].

To improve the fitting accuracy of range history under nonlinear trajectories, the main methods can be divided into three categories. The first category starts from a mathematical perspective and directly fits the range history using Taylor series expansion, such as the fourth-order range model (FORM) [19,20,21,22] and fifth-order range model [23]. The second category uses motion compensation methods similar to airborne SAR, compensating for the differences between nonlinear trajectories and straight-line trajectories as motion errors, and then modeling using the CHRE or ESRM [12,24]. The third category improves the hyperbolic range model. For example, the advanced hyperbolic range equation (AHRE) compensates for high-order term errors by adding the linear term [25], and the modified ESRM (MESRM) [26,27] improves the ESRM by considering the equivalent radar acceleration. MESRM partially compensates for high-order terms based on accurate fitting of the fourth-order term of the actual range history. The EARM [28] and SEARM [29,30] models also improve CHRE and ESRM by considering the velocity’s azimuth variation and adding acceleration. These models also compensate for second-order, third-order, and fourth-order range error terms to accurately fit the real range history. The third category has improved accuracy compared to FORM. In the design process of imaging algorithms, the principle of stationary phase (POSP) [14] and the method of series reversion (MSR) [31] are generally used to derive the frequency domain expression of the echo signal. Therefore, in addition to considering the fitting accuracy of the actual range history, the design of the range model also needs to consider the design of the imaging algorithm. As the foundation of imaging algorithm design, the range model determines the difficulty of subsequent decoupling of range and azimuth.

Based on the above considerations, this paper first models the spatial geometry of spaceborne SAR, analyzes it based on the two-body orbit model and Earth ellipsoid model, and deduces the motion state vectors and Doppler parameters of the satellite and ground targets in detail. It obtains an accurate coordinate numerical model (ACNM) as the real range history. Then, from the perspective of Doppler parameters, it analyzes the fitting accuracy of the range history under nonlinear trajectories by existing range models. Combining the simplicity of the traditional ESRM and the accuracy of the FORM, this paper proposes an advanced ESRM (AESRM). AESRM maintains the original hyperbolic equation and introduces third-order and fourth-order error terms to accurately fit the fourth-order term of the actual range history and further compensate for the high-order term of range history. The derived two-dimensional frequency spectrum form is more concise and facilitates imaging algorithm design. Finally, the effectiveness of the proposed model is verified through experimental simulations.

The rest of this paper is organized as follows: Section 2 introduces the SAR signal model and range model. Section 3 models and solves the spatial geometry of spaceborne SAR. Section 4 analyzes the range modeling of nonlinear trajectories from the perspective of Doppler parameters and proposes AESRM. Then, Section 5 analyzes the key parameters of the satellite and range model errors through simulations, and verifies the model’s effectiveness through point target simulations. Finally, the conclusion is given in Section 6.

## 2. Signal Model and Range Model for SAR

### 2.1. SAR Signal Model

**Figure 1:**
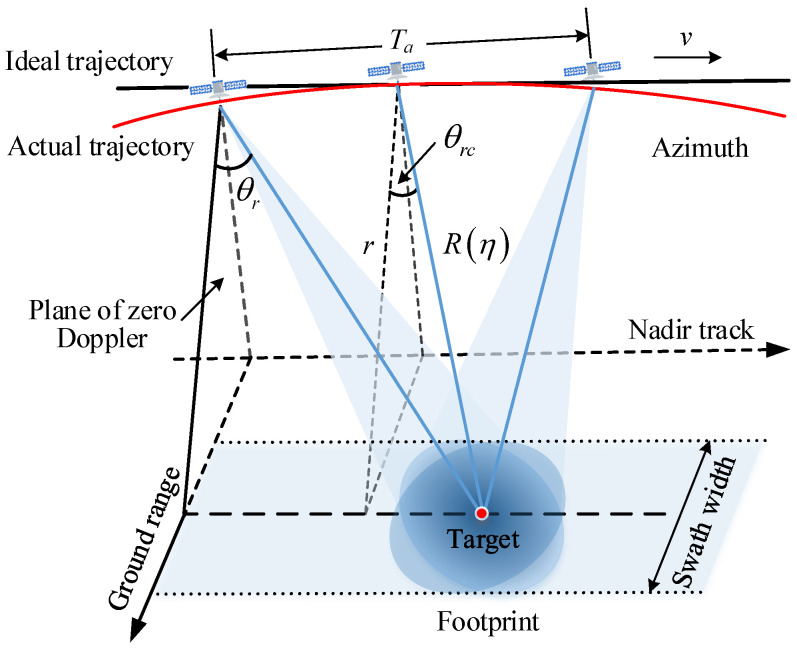
Geometry illustration for spaceborne SAR data acquisition in spotlight mode.

SAR imaging technology utilizes pulse compression to achievehigh-range resolution and coherent accumulation of echoes in the azimuth direction, resulting in high azimuth resolution [1]. The geometric relationship between the radar position and the ground coverage in spotlight mode during spaceborne SAR data acquisition is illustrated in Figure 1. In this figure, R(η) represents the slant range between the radar and the target, whereas *r* is the slant range of closest approach. The squint angle, $\theta_{r}$, varies with the relative position between the radar and the target, whereas $\theta_{rc}$ represents the squint angle at the moment of synthetic aperture center [14]. The radar adjusts the antenna beam to ensure continuous target illumination during the synthetic aperture time $T_{a}$. However, as the resolution increases, $T_{a}$ also increases, and the actual trajectory of the radar cannot be accurately represented as a straight-line motion at a constant velocity *v* [32], leading to defocusing effects in SAR data processing.

The radar transmits a linear frequency modulation (LFM) signal in the range direction, and the received signal is obtained after propagating through the two-way range. The demodulated baseband echo signal from a single point target can be expressed as [1,2]

(1) $$\begin{array}{c} s_{r}\left(\tau ,\eta \right)=A_{0}w_{r}\left(\tau -\frac{2R(\eta )}{c}\right)w_{a}\left(\frac{\eta -\eta_{c}}{T_{a}}\right)\exp\left(-j4\pi \frac{R(\eta )}{\lambda }\right)\exp\left(j\pi K_{r}{\left(\tau -\frac{2R(\eta )}{c}\right)}^{2}\right), \end{array}$$

where $A_{0}$ is a complex constant, τ and η represent the range and azimuth time, respectively, $\eta_{c}$ denotes the center moment of the synthetic aperture time, $w_{r}\left(\cdot \right)$ and $w_{a}\left(\cdot \right)$ are the range and azimuth envelopes, *c* is the speed of light, λ is the radar carrier wavelength, and $K_{r}$ is the FM rate of the transmitted pulse chirp [32].

The phase history of the first term in Equation (1) characterizes the variation in the received signal and represents the change in distance between the radar and the target. The accuracy of modeling the slant range R(η) directly impacts the quality of range and azimuth focusing of the target.

### 2.2. Range Model

The slant range, which varies with azimuth time, is a crucial parameter in SAR signal processing and is commonly described using the range equation or range model. The variation in slant range introduces two effects: phase modulation between different pulses and range cell migration caused by the distortion of echo data. The former is fundamental to the operation of SAR, whereas the latter is a fundamental issue in SAR signal processing [14].

Due to the short distance between airborne SAR and the target, and the rotation of the aircraft with respect to the Earth’s atmosphere, the pointing direction of the beam remains unchanged relative to the flight direction. In the analysis of the motion model, the trajectory can be approximated as a straight line. According to the Pythagorean theorem, the variation in the range between the radar and the target can be described using hyperbolic equations, which leads to the conventional hyperbolic range Equation (CHRE) [14] given by

(2) $$R_{\mathrm{CHRE}}\left(\eta \right)=\sqrt{r^{2}+v^{2}\eta^{2}},$$

where *r* represents the slant range at the closest approach between the radar and the target, i.e., the shortest range, and *v* denotes the radar velocity along the straight trajectory.

**Figure 2:**
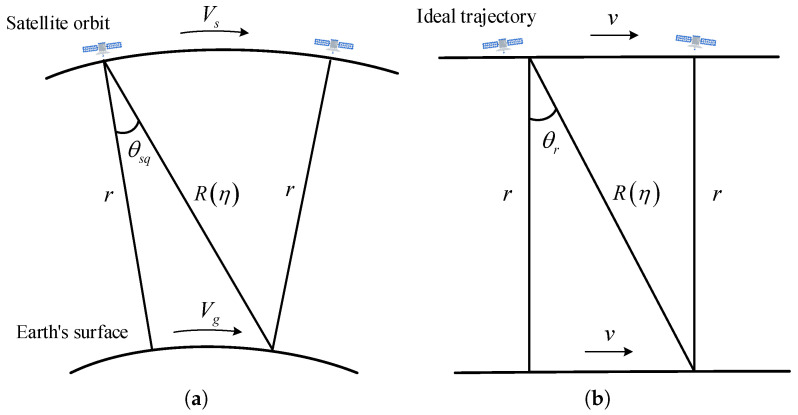
Approximation of curved Earth geometry by rectilinear geometry. (**a**) Curved Earth geometry. (**b**) Rectilinear geometry.

For low-resolution spaceborne SAR, the orbit can be approximated as a straight line using equivalent radar velocity *v* and equivalent squint angle $\theta_{rc}$. To facilitate a better understanding of the approximation of curved Earth geometry by rectilinear geometry, Figure 2 presents a schematic illustration of spaceborne SAR performance under both curved geometry and rectilinear geometry [14]. The satellite moves with a tangential velocity $V_{s}$, whereas the movement speed of the beam coverage area along the ground is represented as $V_{g}$.

However, due to factors such as the curvature of the Earth and its rotation, even in the broadside mode, there exists a certain amount of squint angle. Therefore, it is generally represented by an equivalent squint range model (ESRM) [26], given as

(3) $$\begin{array}{cc} R_{\mathrm{ESRM}}\left(\eta \right) & =\sqrt{{\left(r_{c}\cos\theta_{\mathrm{rc}}\right)}^{2}+{\left(v\eta -r_{c}\sin\theta_{\mathrm{rc}}\right)}^{2}}\\ \; & =\sqrt{{r_{c}}^{2}+v^{2}\eta^{2}-2r_{c}v\eta \sin\theta_{\mathrm{rc}}}\;, \end{array}$$

where $r_{c}$ represents the slant range between the platform and the target at the center of synthetic aperture time. These parameters vary with the motion of the satellite, Earth rotation, and different orbital positions, resulting in varying ranges that need to be calculated using Doppler parameters.

The range model plays a critical role in SAR data processing as it forms the basis for obtaining high-resolution images. However, the range model is also a significant factor leading to distortion in the raw echo data and range–azimuth coupling, and its accuracy greatly affects the quality of subsequent imaging results. In most cases, we assume that the radar moves in a straight line and the target remains stationary to simplify the solution process. However, in high-resolution imaging scenarios, the platform’s trajectory exhibits nonlinear characteristics, and the ESRM cannot accurately describe the variations in range. To accurately describe the variation in slant range, precise modeling of the spatial geometry of the radar and target is required.

## 3. Space Geometry Modeling of Spaceborne SAR

Spaceborne SAR achieves high azimuth resolution by accumulating coherent pulses in the azimuth direction with the satellite platform’s motion. This accumulation process requires considering the effect of platform trajectory and satellite–Earth motion parameters on the range model, which is crucial for SAR data processing. In this section, we focus on the two-body orbit model and the Earth ellipsoid model to calculate the state vectors of satellite–Earth motion and Doppler parameters through spatial geometric analysis. Finally, we derive an accurate coordinate numerical model (ACNM) of slant range based on the position vectors of the satellite and target.

### 3.1. Two-Body Orbit Model

**Figure 3:**
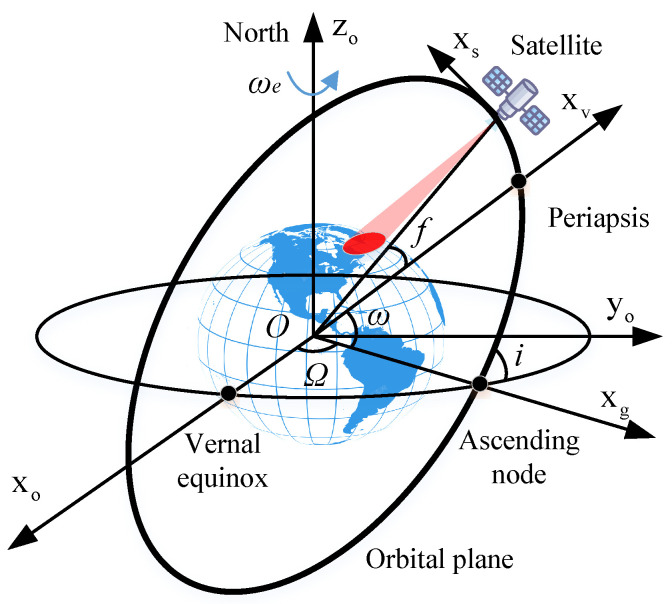
Space geometry of spaceborne SAR in ECI coordinate system.

The primary influence on a satellite’s orbit around the Earth is the gravitational interaction between the satellite and the Earth itself. Thus, a simplified two-body orbit model suffices to characterize the satellite’s trajectory [32,33,34]. Figure 3 depicts the spatial geometry of spaceborne SAR within the Earth-centered inertial coordinate system (ECI) [32].

The satellite’s position along its elliptical orbit is defined by six orbital elements: the semi-major axis *a*, eccentricity *e*, inclination *i*, right ascension of the ascending node (RAAN) Ω, argument of perigee ω, and true anomaly *f* [32,34].

In the absence of external perturbation forces, Kepler’s three laws can be employed to compute the satellite’s orbit. The equation describing the satellite’s orbit [32,33] is

(4) $$R_{s}=\frac{a\left(1-e^{2}\right)}{1+e\cos f}.$$

Since the Earth is not a perfect ellipsoid, the WGS-84 model is generally used to describe the shape and position of the Earth, which has been widely used in geodesy and satellite navigation. The position of the target on the Earth ${\left[x_{t},y_{t},z_{t}\right]}^{T}$ can be determined by

(5) $$\frac{x_{t}^{2}}{R_{e}^{2}}+\frac{y_{t}^{2}}{R_{e}^{2}}+\frac{z_{t}^{2}}{R_{p}^{2}}=1.$$

where $R_{e}$ and $R_{p}$ are the equatorial radius and polar radius of the Earth, respectively. For the sake of analysis convenience, we did not take into account the influence of target elevation in Equation (5), and subsequent research was conducted based on the assumption of flat terrain.

### 3.2. Space Coordinate Systems and Transformation Relationships

To effectively simulate the functioning of spaceborne SAR systems, it is fundamental to ascertain the movement patterns of both satellites and terrestrial targets. The geometric construction of SAR systems is intricately linked to eight distinct coordinate frameworks, namely, Earth-centered rotating coordinate system (ECR) $E_{g}$, Earth-centered inertial coordinate system (ECI) $E_{o}$, orbital plane coordinate system $E_{v}$, satellite platform coordinate system $E_{r}$, satellite body coordinate system $E_{e}$, antenna coordinate system $E_{a}$, satellite scene coordinate system $E_{c}$, and ground coordinate system $E_{f}$ [32,33,34,35].

**Figure 4:**
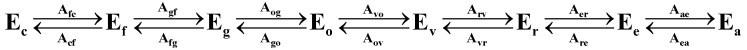
Transformation relationships and transformation matrix of spatial coordinate systems.

The process of transitioning among these diverse coordinate frameworks is vital for the geometric modeling of spaceborne SAR. This transition chiefly encompasses maneuvers of translation and rotation. Figure 4 illustrates these conversion processes, highlighting the transformation relations and the corresponding interconversion matrices between the coordinate systems, with comprehensive definitions provided in [32].

In the spatial geometric modeling analysis and parameter estimation of spaceborne SAR, selecting appropriate coordinate systems for different scenarios is crucial as it effectively reduces the complexity of parameter analysis and solution.

### 3.3. Satellite–Earth Motion State Vectors

With the improvement in the resolution of spaceborne SAR, traditional circular orbit approximations may introduce significant errors, necessitating the consideration of more precise orbit models. Factors such as elliptical orbits and Earth rotation become critically important in modeling the motion trajectory of spaceborne SAR. Accurately determining the motion state vectors of satellites and targets forms the basis of modeling the motion trajectory of spaceborne SAR. These vectors are used to compute essential information such as SAR system operational parameters, Doppler parameters, and range model, ensuring high-quality imaging results.

#### 3.3.1. Satellite Motion State Vectors

The satellite motion vector can be conveniently calculated in the orbit plane coordinate system $E_{v}$ through the *f* and $R_{s}$. Meanwhile, the position and velocity vectors of the satellite in the ECI coordinate system, denoted as $r_{s}$ and $v_{s}$ [34,35], respectively, can be obtained through coordinate transformation,

(6) $$r_{s}=A_{\mathrm{ov}}\left(R_{s}{\left[\cos f,\sin f,0\right]}^{T}\right),$$

(7) $$v_{s}=A_{\mathrm{ov}}\left(\sqrt{\frac{\mu }{a\left(1-e^{2}\right)}}{\left[-\sin f,e+\cos f,0\right]}^{T}\right).$$

where the gravitational constant μ is $3.98696\times 10^{14}\;m^{3}/s^{2}$.

The acceleration vector of the satellite can be obtained by differentiating Equation (7) as

(8) $$a_{s}=A_{\mathrm{ov}}\left(\frac{\mu }{R_{s}^{2}}{\left[-\cos f,-\sin f,0\right]}^{T}\right).$$

The instantaneous angular velocity of the satellite and its first derivative are given by

(9) $$\omega_{s}=f^{\prime }=\frac{\sqrt{\mu a(1-e^{2})}}{R_{s}^{2}},$$

(10) $$\omega_{s}^{\prime }=-\frac{2\sqrt{\mu a(1-e^{2})}}{R_{s}^{3}}R_{s}^{\prime }.$$

Additionally, the rate of change in the satellite’s polar radius is denoted as $R_{s}^{\prime }=v_{s}\cdot r_{s}/R_{s}$.

The first and second derivatives of the satellite’s acceleration can be derived from Equation (8) as

(11) $$a_{s}^{\prime }=A_{\mathrm{ov}}\left(\frac{2\mu }{R_{s}^{3}}R_{s}^{\prime }{\left[\cos f,\sin f,0\right]}^{T}+\frac{\mu }{R_{s}^{2}}\omega_{s}{\left[\sin f,-\cos f,0\right]}^{T}\right),$$

(12) $$a_{s}^{\prime \prime }=A_{\mathrm{ov}}\left(\begin{array}{cc} \; & \left(\frac{2\mu }{R_{s}^{3}}R_{s}^{\prime \prime }-\frac{6\mu }{R_{s}^{4}}R_{s}^{\prime 2}+\frac{\mu }{R_{s}^{2}}\omega_{s}^{2}\right){\left[\cos f,\sin f,0\right]}^{T}\\ \; & +\left(\frac{4\mu }{R_{s}^{3}}R_{s}^{\prime }\omega_{s}-\frac{\mu }{R_{s}^{2}}\omega_{s}^{\prime }\right){\left[-\sin f,\cos f,0\right]}^{T} \end{array}\right).$$

Moreover, the second derivative of the satellite’s polar radius is expressed as $R_{s}^{\prime \prime }=\left(R_{s}\left(a_{s}\cdot r_{s}+v_{s}^{2}\right)-\left(v_{s}\cdot r_{s}\right)R_{s}^{\prime }\right)/R_{s}^{2}$.

The aforementioned state vectors of satellite motion can be further mapped into other coordinate systems through spatial coordinate transformation. It is important to note that due to the time-dependent nature of the transformation matrix from the ECI coordinate system to the ECR coordinate system, except for the position vector, the conversion of other motion state vectors cannot be achieved simply by matrix multiplication.

#### 3.3.2. Determination of Antenna Beam Aiming Point

When a satellite operates along an elliptical orbit, the spaceborne SAR beam scans over an area on the Earth’s surface, which is referred to as the beam coverage area or footprint. Within the beam coverage area, the intersection of the SAR beam’s centerline and the Earth’s surface forms a series of points, each referred to as an aiming point of the beam.

In SAR imaging, the center position of the imaging swath is determined by the beam aiming points, which in turn determines the central region of SAR imaging. By precisely controlling the beam aiming points, different ground areas can be selected for imaging, enabling high-resolution observation and imaging of target areas.

The parameter to be solved is the distance $y_{a}$ from the satellite to the aiming point, which has coordinates ${\left[0,y_{a},0\right]}^{T}$ in the antenna coordinate system $E_{a}$. Let the position vector of the antenna phase center in the satellite body coordinate system $E_{e}$ be $r_{\mathrm{ea}}$. The position vector of the antenna phase center in the ECR coordinate system $E_{g}$ [34,36] is

(13) $$r_{\mathrm{ga}}=A_{\mathrm{go}}A_{\mathrm{ov}}\left(A_{\mathrm{vr}}A_{\mathrm{re}}\left(A_{\mathrm{ea}}{\left[0,y_{a},0\right]}^{T}+r_{\mathrm{ea}}\right)+r_{s}\right).$$

Substituting the obtained position vector $r_{\mathrm{ga}}$ into the smooth ellipsoidal Earth model with Equation (5), the minimum of the two solutions for $y_{a}$ is obtained. If the solution is a complex number, it suggests that the centerline of the beam deviates from the Earth’s surface.

#### 3.3.3. Target Motion State Vectors

Consideration of the Earth’s rotation effect makes it more convenient to determine the motion state vectors of ground targets in the ECR coordinate system $E_{g}$. In SAR raw data simulation, the position of the target point is usually represented in the satellite scene coordinate system $E_{c}$, with its origin at the center of the scene.

Typically, the SAR system takes the beam aiming point at the imaging center as the scene center, which also serves as the center of the imaging swath. This point serves as the reference point in the $E_{c}$. By utilizing Equation (13) to obtain the coordinates of the scene center in the $E_{g}$, the transformation matrices $A_{\mathrm{fc}}$ and $A_{\mathrm{gf}}$ can be used to transform the coordinates of other targets within the scene to $E_{g}$ [32]. The transformation relationship is

(14) $$r_{\mathrm{gt}}=A_{\mathrm{gf}}A_{\mathrm{fc}}r_{\mathrm{ct}}+r_{\mathrm{ga}0},$$

where $r_{\mathrm{ct}}$ represents the position vector of other targets in the $E_{c}$, whereas $r_{\mathrm{ga}0}$ denotes the position vector of the beam aiming point in the $E_{g}$ at the imaging center time.

In the ECR coordinate system, all ground targets are stationary, and their position vectors remain unchanged, resulting in their velocity vector being $v_{\mathrm{gt}}=0$.

In the ECI coordinate system, ground targets rotate with the Earth, and thus the position vector [21] is

(15) $$r_{t}=R_{\mathrm{ta}}\cos\theta_{\mathrm{lat}}{\left[\cos\theta_{\mathrm{lon}}\left(t\right),\sin\theta_{\mathrm{lon}}\left(t\right),\tan\theta_{\mathrm{lat}}\right]}^{T},$$

where $R_{\mathrm{ta}}$ represents the Earth radius at the latitude circle where the target is located, and $\theta_{\mathrm{lon}}$ and $\theta_{\mathrm{lat}}$, respectively, denote the longitude and latitude of the target. Moreover, $\theta_{\mathrm{lon}}\left(t\right)=\theta_{\mathrm{lon}0}+\omega_{e}t$, where *t* represents the time relative to the initial longitude angle $\theta_{\mathrm{lon}0}$ of the target and $\omega_{e}$ is the angular velocity of the Earth’s rotation.

By sequentially differentiating Equation (15), the velocity and acceleration vectors of the ground target can be obtained as

(16) $$v_{t}=R_{\mathrm{ta}}\omega_{e}\cos\theta_{\mathrm{lat}}{\left[-\sin\theta_{\mathrm{lon}}\left(t\right),\cos\theta_{\mathrm{lon}}\left(t\right),0\right]}^{T},$$

(17) $$a_{t}=-R_{\mathrm{ta}}\omega_{e}^{2}\cos\theta_{\mathrm{lat}}{\left[\cos\theta_{\mathrm{lon}}\left(t\right),\sin\theta_{\mathrm{lon}}\left(t\right),0\right]}^{T}.$$

Subsequently, by sequentially differentiating Equation (17), the first- and second-order acceleration vectors of the ground target can be derived as

(18) $$a_{t}^{\prime }=-R_{\mathrm{ta}}\omega_{e}^{3}\cos\theta_{\mathrm{lat}}{\left[-\sin\theta_{\mathrm{lon}}\left(t\right),\cos\theta_{\mathrm{lon}}\left(t\right),0\right]}^{T},$$

(19) $$a_{t}^{\prime \prime }=R_{\mathrm{ta}}\omega_{e}^{4}\cos\theta_{\mathrm{lat}}{\left[\cos\theta_{\mathrm{lon}}\left(t\right),\sin\theta_{\mathrm{lon}}\left(t\right),0\right]}^{T}.$$

The above values represent the motion state vectors of ground targets in the ECI coordinate system, and the values in other coordinate systems can be obtained through spatial coordinate transformation [32].

### 3.4. Calculation of Doppler Parameters

The Doppler parameters of spaceborne SAR are crucial for signal modeling and data processing, which require precise analysis of the relative motion between the satellite and the ground.

To reduce the complexity of parameter estimation, the relative motion state vector between the satellite and the ground target is uniformly solved in the ECI coordinate system. The 1st- to 5th-order motion state vectors of the satellite and ground target were introduced in the previous section, from which the relative motion state vector between the satellite and the target can be correspondingly derived, for example,

(20) $$r=\left|r_{\mathrm{st}}\right|=\left|r_{s}-r_{t}\right|,$$

where the subscript “st” denotes the relative motion state vector between the satellite and the target.

The 1st- to 4th-order Doppler parameters can be obtained by differentiating the relative position vector [34], which are

(21) $$f_{\mathrm{dc}}=-\frac{2}{\lambda }r^{\prime }=-\frac{2}{\lambda }\frac{r_{\mathrm{st}}\cdot v_{\mathrm{st}}}{r},$$

(22) $$f_{r}=-\frac{2}{\lambda }r^{\prime \prime }=-\frac{2}{\lambda }\left(\frac{v_{\mathrm{st}}\cdot v_{\mathrm{st}}}{r}+\frac{a_{\mathrm{st}}\cdot r_{\mathrm{st}}}{r}-\frac{\lambda^{2}f_{\mathrm{dc}}^{2}}{4r}\right),$$

(23) $$f_{2r}=-\frac{2}{\lambda }r^{\left(3\right)}=-\frac{2}{\lambda }\left(\frac{3a_{\mathrm{st}}\cdot v_{\mathrm{st}}}{r}+\frac{a_{\mathrm{st}}^{\prime }\cdot r_{\mathrm{st}}}{r}-\frac{3\lambda^{2}f_{\mathrm{dc}}f_{r}}{4r}\right),$$

(24) $$f_{3r}=-\frac{2}{\lambda }r^{\left(4\right)}=-\frac{2}{\lambda }\left(\frac{3a_{\mathrm{st}}\cdot a_{\mathrm{st}}}{r}+\frac{4a_{\mathrm{st}}^{\prime }\cdot v_{\mathrm{st}}}{r}+\frac{a_{\mathrm{st}}^{\prime \prime }\cdot r_{\mathrm{st}}}{r}-\frac{3\lambda^{2}f_{r}^{2}}{4r}-\frac{\lambda^{2}f_{\mathrm{dc}}f_{2r}}{4r}\right).$$

The above derivation of spaceborne SAR Doppler parameters does not rely on any assumptions and is accurate under the two-body orbit model. The analysis can also be applied to spaceborne SAR analysis at other orbital heights.

### 3.5. Accurate Coordinate Numerical Model

By modeling the spatial geometry of spaceborne SAR, we can obtain the motion state vector of the satellite and the target. By calculating the position vectors of the satellite and the ground target at each azimuth time, the change in slant range can be accurately calculated. The slant range obtained by this method is called the accurate coordinate numerical model (ACNM) [21].

The position vector of the target point in the ECR coordinate system can be obtained through Equations (13) and (14), and after coordinate transformation, the position vector of the target in the ECI coordinate system can be obtained as

(25) $$r_{\mathrm{ot}}=A_{\mathrm{og}}r_{\mathrm{gt}}={\left[x_{t}\left(\eta \right),y_{t}\left(\eta \right),z_{t}\left(\eta \right)\right]}^{T}.$$

Assuming that at the azimuth center time $\eta_{c}$, the position coordinates of the target point are represented as ${\left[x_{t}\left(\eta_{c}\right),y_{t}\left(\eta_{c}\right),z_{t}\left(\eta_{c}\right)\right]}^{T}$, we can calculate the local latitude and local Earth radius of the target point in the ECI coordinate system [21], represented by

(26) $$\theta_{\mathrm{latc}}=\arctan\left(z_{t}\left(\eta_{c}\right)/\sqrt{x_{t}^{2}\left(\eta_{c}\right)+y_{t}^{2}\left(\eta_{c}\right)}\right),$$

(27) $$\theta_{\mathrm{lonc}}=\left\{\begin{array}{c} \arctan\left(y_{t}\left(\eta_{c}\right)/x_{t}\left(\eta_{c}\right)\right),x_{t}\left(\eta_{c}\right)>0\\ \arctan\left(y_{t}\left(\eta_{c}\right)/x_{t}\left(\eta_{c}\right)\right)+\pi ,x_{t}\left(\eta_{c}\right)<0,y_{t}\left(\eta_{c}\right)>0\\ \arctan\left(y_{t}\left(\eta_{c}\right)/x_{t}\left(\eta_{c}\right)\right)-\pi ,x_{t}\left(\eta_{c}\right)<0,y_{t}\left(\eta_{c}\right)<0 \end{array}\right.,$$

(28) $$R_{\mathrm{tac}}=\sqrt{\frac{R_{e}^{2}R_{p}^{2}}{{\left(R_{p}\cos\theta_{\mathrm{latc}}\right)}^{2}+{\left(R_{e}\sin\theta_{\mathrm{latc}}\right)}^{2}}}\;.$$

In the ECI coordinate system, the ground target moves with the rotation of the Earth. Due to the symmetry of the ellipsoid model, the local Earth radius and latitude of the target point remain unchanged, and only the longitude changes over time. According to Equation (15), the position vector of the target point can be calculated as

(29) $$\left[\begin{array}{c} x_{t}\left(\eta \right)\\ y_{t}\left(\eta \right)\\ z_{t}\left(\eta \right) \end{array}\right]=\left[\begin{array}{c} R_{\mathrm{tac}}\cos\theta_{\mathrm{latc}}\cos\theta_{\mathrm{lon}}\left(\eta \right)\\ R_{\mathrm{tac}}\cos\theta_{\mathrm{latc}}\sin\theta_{\mathrm{lon}}\left(\eta \right)\\ R_{\mathrm{tac}}\sin\theta_{\mathrm{latc}} \end{array}\right],$$

where the change in longitude with azimuth time η can be represented as $\theta_{\mathrm{lon}}\left(\eta \right)=\theta_{\mathrm{lonc}}+\omega_{e}\left(\eta -\eta_{c}\right)$.

The position vector of the satellite in the ECI coordinate system is calculated by Equation (6), i.e., $r_{s}={\left[x_{s}\left(\eta \right),y_{s}\left(\eta \right),z_{s}\left(\eta \right)\right]}^{T}$. Then, the real range between the satellite and the target is

(30) $$R_{\mathrm{ACNM}}\left(\eta \right)=\sqrt{{\left(x_{t}\left(\eta \right)-x_{s}\left(\eta \right)\right)}^{2}+{\left(y_{t}\left(\eta \right)-y_{s}\left(\eta \right)\right)}^{2}+{\left(z_{t}\left(\eta \right)-z_{s}\left(\eta \right)\right)}^{2}}.$$

In order to accurately express the variation in slant range, it is also possible to represent the relative motion state vector between the radar and the target mathematically. By performing a Taylor expansion of slant range at azimuth time zero (generally at the midpoint of synthetic aperture time) and using Equations (21)–(24) [34], the expression is

(31) $$\begin{array}{cc} R_{\mathrm{ACNM}}\left(\eta \right) & =r_{c}+r^{\prime }\eta +\frac{1}{2!}r^{\prime \prime }\eta^{2}+\frac{1}{3!}r^{\left(3\right)}\eta^{3}+\frac{1}{4!}r^{\left(4\right)}\eta^{4}+\cdots \\ \; & =r_{c}-\frac{\lambda }{2}f_{\mathrm{dc}}\eta -\frac{\lambda }{4}f_{r}\eta^{2}-\frac{\lambda }{12}f_{2r}\eta^{3}-\frac{\lambda }{48}f_{3r}\eta^{4}+\cdots . \end{array}$$

By modeling the spatial geometry of spaceborne SAR, combining the two-body orbit model and the Earth ellipsoid model, we can obtain the motion state vector of the satellite and the ground target. The ACNM obtained by calculating the position vectors has high accuracy, which can be used as the evaluation standard for the fitting accuracy of other range models.

## 4. Range Model of Nonlinear Trajectory of Spaceborne SAR

Building on the precise geometric modeling of satellite and ground targets discussed in the previous section, this section focuses on different range models for fitting the range history under the nonlinear trajectory of the spaceborne SAR from the perspective of Doppler parameters. In addition, the fitting accuracy of different range models is analyzed.

### 4.1. Conventional Hyperbolic Range Equation

Since only the equivalent radar velocity and equivalent squint angle parameters need to be solved in the ESRM, we can perform a Taylor expansion of Equation (3) at zero azimuth time and retain the second-order term

(32) $$R_{\mathrm{ESRMs}}\left(\eta \right)=r_{c}-v\sin\theta_{\mathrm{rc}}\eta +\frac{1}{2}\frac{v^{2}\cos^{2}\theta_{\mathrm{rc}}}{r_{c}}\eta^{2}.$$

By solving Equations (31) and (32) simultaneously, the solution can be obtained

(33) $$\left\{\begin{array}{c} v=\sqrt{{\left(\frac{\lambda f_{\mathrm{dc}}}{2}\right)}^{2}-\frac{\lambda r_{c}f_{r}}{2}}\\ \theta_{\mathrm{rc}}=\arcsin\left(\frac{\lambda f_{\mathrm{dc}}}{2v}\right) \end{array}\right..$$

The CHRE utilizes only the Doppler centroid and Doppler FM rate parameter to fit the range history. However, when the resolution of the SAR system is high, the synthetic aperture time for targets becomes longer, and the effects of orbit curvature, Earth rotation, and satellite velocity variations become more prominent. This leads to a more noticeable nonlinearity in the satellite trajectory. In such circumstances, approximating the trajectory as a uniform straight line would introduce significant errors and negatively impact the azimuth focusing performance.

### 4.2. Motion Compensation Range Model

As the resolution of spaceborne SAR reaches the sub-meter level, the increasing synthetic aperture time leads to a growing error of CHRE caused by the curvature of the trajectory, resulting in phase errors exceeding π/4 and severely affecting the focusing quality [37].

In order to meet the imaging requirements of high-resolution SAR, a motion compensation range model (MCRM) is proposed in [12] to correct errors introduced by the curvature of the orbit. This model compensates for these errors by making the motion trajectory approach a straight line, allowing for better utilization of CHRE and mature traditional frequency-domain algorithms.

Similar to the two-step motion compensation method used in airborne SAR [12,24], the slant range error caused by orbit curvature can be divided into two components: range-independent and range-dependent. These components are compensated for separately using coarse compensation and fine compensation, respectively. The coarse compensation is based on correcting the range history of the center target in the scene, whereas the compensation function used in the azimuth time domain can be expressed as

(34) $$H_{\mathrm{OCO}1}\left(\eta ,f_{\tau };r_{\mathrm{ref}}\right)=\exp\left(j\frac{4\pi }{c}\left(f_{0}+f_{\tau }\right)\Delta R_{\mathrm{ref}}\left(\eta \right)\right),$$

where $\Delta R_{\mathrm{ref}}\left(\eta \right)$ represents the slant range fitting error for the scene center target, given by $\left(R_{\mathrm{st}},\mathrm{ref}-R_{\mathrm{ref}}\left(\eta \right)\right)$, where $R_{\mathrm{st}},\mathrm{ref}$ is the true range history of the scene center target obtained from orbit parameters, and $R_{\mathrm{ref}}\left(\eta \right)$ is the hyperbolic slant range history of the scene center target calculated using Equation (3).

The compensation mentioned above only achieves complete compensation for the scene center target, whereas range-dependent errors still exist for other targets [12]. Therefore, a fine compensation step that is range-dependent is required to compensate for these errors, namely,

(35) $$H_{\mathrm{OCO}2}\left(\eta ,r\right)=\exp\left(j\frac{4\pi }{\lambda }\left(\Delta R_{(}\eta )-\Delta R_{\mathrm{ref}}\left(\eta \right)\right)\right),$$

where ΔR(η) represents the hyperbolic slant range fitting error for other targets. For larger errors, additional range interpolation may be required to correct the range-dependent offset. However, due to the relatively large distance of spaceborne SAR and the small imaging scene in spotlight mode, range-dependency can usually be ignored [12].

The two-step motion compensation method is a simple and effective way to compensate for the slant range errors caused by trajectory curvature. However, in the case of wide swath or ultra-high resolution, the compensation effect for targets at the edge of the scene is limited, and block processing or higher-precision range models may be needed [21,26,27,28].

### 4.3. Fourth-Order Range Model

To more accurately fit range history under nonlinear trajectories, we can utilize Taylor series expansions to fit the variations in the slant range. The higher the order of expansion, the higher the accuracy of the fit [23]. However, as the expansion order increases, the design and processing difficulty of subsequent imaging algorithms also increases. Moreover, the accuracy improvement becomes limited. Therefore, in practical applications, the commonly used model is the fourth-order range model (FORM) [20,21,22], which is formulated as

(36) $$R_{\mathrm{FORM}}\left(\eta \right)=r_{c}+k_{1}\eta +k_{2}\eta^{2}+k_{3}\eta^{3}+k_{4}\eta^{4}.$$

The coefficients in FORM can be obtained from Equation (31),

(37) $$\left\{\begin{array}{c} k_{1}=-\frac{\lambda }{2}f_{\mathrm{dc}}\\ k_{2}=-\frac{\lambda }{4}f_{r}\\ k_{3}=-\frac{\lambda }{12}f_{2r}\\ k_{4}=-\frac{\lambda }{48}f_{3r} \end{array}\right..$$

In contrast to alternative range models, FORM achieves a higher level of accuracy in fitting third- and fourth-order terms through the integration of four Doppler parameters. This enhances the precision of range history fitting, particularly in the context of nonlinear trajectories.

### 4.4. Improved Range Model Based on CHRE

Due to the nonlinear characteristics of trajectory fitting in high-resolution spaceborne SAR, whereas CHRE can only fit linear trajectories, the MCRM is unable to meet the requirements of the wide azimuth swath. Therefore, in order to further improve the accuracy on the basis of FORM, several improved range models have been proposed by modifying CHRE. In the following sections, we will analyze and introduce these improvements from the perspective of Doppler parameter angles.

#### 4.4.1. Advanced Hyperbolic Range Equation

The use of the CHRE for slant range fitting only relies on two parameters: the Doppler centroid and Doppler FM rate. This limits the approximation of the true range history to second order [14]. In order to improve the accuracy of slant range fitting, the advanced hyperbolic range equation (AHRE), which compensates for residual higher-order term errors by introducing a linear term, has been proposed [25]. It can be expressed as

(38) $$R_{\mathrm{AHRE}}\left(\eta \right)=\sqrt{{r_{c}}^{2}+v^{2}\eta^{2}-2r_{c}v\eta \sin\theta_{\mathrm{rc}}}+\Delta_{l}\eta ,$$

where Δr represents the coefficient of the linear term added to AHRE based on CHRE to compensate for errors.

Since AHRE involves solving three parameters, we can perform a Taylor expansion of Equation (38) at zero azimuth time while retaining the third-order term, resulting in

(39) $$R_{\mathrm{AHREs}}\left(\eta \right)=r_{c}-\left(vs.\sin\theta_{\mathrm{rc}}-\Delta_{l}\right)\eta +\frac{v^{2}\cos^{2}\theta_{\mathrm{rc}}}{2r_{c}}\eta^{2}+\frac{v^{3}\cos^{2}\theta_{\mathrm{rc}}\sin\theta_{\mathrm{rc}}}{2r_{c}^{2}}\eta^{3}.$$

By simultaneously solving Equations (31) and (39), we obtain

(40) $$\left\{\begin{array}{c} v=\sqrt{{\left(\frac{r_{c}f_{2r}}{3f_{r}}\right)}^{2}-\frac{\lambda r_{c}f_{r}}{2}}\\ \theta_{\mathrm{rc}}=\arcsin\left(\frac{r_{c}f_{2r}}{3vf_{r}}\right)\\ \Delta_{l}=-\frac{\lambda f_{\mathrm{dc}}}{2}+\frac{r_{c}f_{2r}}{3f_{r}} \end{array}\right..$$

It can be observed that AHRE, compared to CHRE, employs an additional Doppler parameter, allowing for more accurate fitting of nonlinear slant ranges. However, due to only considering the linear part of the error, the accuracy remains limited.

#### 4.4.2. Modified Equivalent Squint Range Model

To enhance the accuracy of the CHRE, the modified ESRM (MESRM) introduces the concept of equivalent radar acceleration [26,27]. The MESRM is formulated as follows:

(41) $$\begin{array}{cc} R_{\mathrm{MESRM}}\left(\eta \right) & =\sqrt{r_{c}^{2}+{\left(v_{0}\eta +\frac{1}{2}a_{r}\eta^{2}\right)}^{2}-2r_{c}\left(v_{0}\eta +\frac{1}{2}a_{r}\eta^{2}\right)\sin\theta_{0}}\\ \; & =\sqrt{r_{c}^{2}+v^{2}\eta^{2}-2r_{c}v\eta \sin\theta_{\mathrm{rc}}+\Delta a_{3}\eta^{3}+\Delta a_{4}\eta^{4}}, \end{array}$$

where $a_{r}$ represents the introduced equivalent radar acceleration in the MESRM. The second equation in Equation (41) is a simplified form for ease of comparison.

The MESRM incorporates two additional parameters, $\Delta a_{3}$ and $\Delta a_{4}$, to the CHRE. By performing a Taylor expansion of Equation (41) at azimuth time zero and retaining the fourth-order term, we obtain:

(42) $$\begin{array}{cc} R_{\mathrm{MESRMs}}\left(\eta \right) & =r_{c}-vs.\sin\theta_{\mathrm{rc}}\eta +\frac{v^{2}\cos^{2}\theta_{\mathrm{rc}}}{2r_{c}}\eta^{2}+\left(\frac{\Delta a_{3}}{2r_{c}}+\frac{v^{3}\cos^{2}\theta_{\mathrm{rc}}\sin\theta_{\mathrm{rc}}}{2r_{c}^{2}}\right)\eta^{3}\\ \; & +\left(\frac{\Delta a_{4}}{2r_{c}}+\frac{\Delta a_{3}v\sin\theta_{\mathrm{rc}}}{2r_{c}^{2}}+\frac{v^{4}\cos^{2}\theta_{\mathrm{rc}}\left(5\sin^{2}\theta_{\mathrm{rc}}-1\right)}{8r_{c}^{3}}\right)\eta^{4}. \end{array}$$

By simultaneously solving Equations (31) and (42), the solution can be obtained,

(43) $$\left\{\begin{array}{c} v=\sqrt{{\left(\frac{\lambda f_{\mathrm{dc}}}{2}\right)}^{2}-\frac{\lambda r_{c}f_{r}}{2}}\\ \theta_{\mathrm{rc}}=\arcsin\left(\frac{\lambda f_{\mathrm{dc}}}{2v}\right)\\ \Delta a_{3}=-\frac{\lambda r_{c}f_{2}r}{6}-\frac{v^{3}\sin\theta_{\mathrm{rc}}\cos^{2}\theta_{\mathrm{rc}}}{r_{c}}\\ \Delta a_{4}=-\frac{\lambda r_{c}f_{3}r}{24}+\frac{v^{4}\cos^{2}\theta_{\mathrm{rc}}}{4r_{c}^{2}}\left(1-5\sin^{2}\theta_{\mathrm{rc}}\right)-\frac{\Delta a_{3}v\sin\theta_{\mathrm{rc}}}{r_{c}} \end{array}\right..$$

The MESRM also utilizes four Doppler parameters for fitting. Compared to the FORM, the MESRM not only accurately fits the fourth-order term but also compensates for some higher-order term errors. This higher accuracy of MESRM in fitting range history allows for longer synthetic aperture times and can be applied to achieve ultra-high-resolution imaging.

#### 4.4.3. Squint Equivalent Acceleration Range Model

To better illustrate the variation in equivalent radar velocity in the azimuth direction, ref. [29] proposes a squint equivalent acceleration range model (SEARM) that considers satellite motion as a constant acceleration straight-line motion centered on a reference target. The SEARM can be expressed as follows:

(44) $$\begin{array}{cc} R_{\mathrm{SEARM}}\left(\eta \right)\; & =\sqrt{{r_{c}}^{2}+{\left(v\eta +\frac{1}{2}a_{r}\eta^{2}\right)}^{2}-2r_{c}\sin\theta_{\mathrm{rc}}\left(v\eta +\frac{1}{2}a_{r}\eta^{2}\right)}\\ \; & +\beta_{2}\eta^{2}+\beta_{3}\eta^{3}+\beta_{4}\eta^{4}, \end{array}$$

where $a_{r}$ represents the introduced equivalent radar acceleration, $\beta_{2}$, $\beta_{3}$, and $\beta_{4}$ are coefficients of the second-, third-, and fourth-order error terms in SEARM. Based on the FORM, residual third- and fourth-order error terms are added, but the parameters are approximated using the center of the scene [28,29,30].

By Taylor expanding Equation (44) at zero azimuth time while retaining the fourth-order term, we have:

(45) $$\begin{array}{cc} R_{\mathrm{SEARMs}}\left(\eta \right) & =r_{c}-vs.\sin\theta_{\mathrm{rc}}\eta +\left(\frac{v^{2}\cos^{2}\theta_{\mathrm{rc}}}{2r_{c}}-\frac{a_{r}\sin\theta_{\mathrm{rc}}}{2}+\beta_{2}\right)\eta^{2}\\ \; & +\left(\frac{a_{r}v\cos^{2}\theta_{\mathrm{rc}}}{2r_{c}}+\frac{v^{3}\cos^{2}\theta_{\mathrm{rc}}\sin\theta_{\mathrm{rc}}}{2r_{c}^{2}}+\beta_{3}\right)\eta^{3}\\ \; & +\left(\frac{a_{r}^{2}\cos^{2}\theta_{\mathrm{rc}}}{8r_{c}}+\frac{3a_{r}v^{2}\cos^{2}\theta_{\mathrm{rc}}\sin\theta_{\mathrm{rc}}}{4r_{c}^{2}}+\frac{v^{4}\cos^{2}\theta_{\mathrm{rc}}\left(5\sin^{2}\theta_{\mathrm{rc}}-1\right)}{8r_{c}^{3}}+\beta_{4}\right)\eta^{4}. \end{array}$$

By comparing with Equation (31), assuming $f_{\mathrm{dc}}=\frac{2v\sin\theta_{\mathrm{rc}}}{\lambda }$ and $f_{r}=-\frac{2v^{2}\cos^{2}\theta_{\mathrm{rc}}}{\lambda r_{c}}$, the solution can be obtained as follows:

(46) $$\left\{\begin{array}{c} v=\sqrt{{\left(\frac{\lambda f_{\mathrm{dc}}}{2}\right)}^{2}-\frac{\lambda r_{c}f_{r}}{2}}\\ \theta_{\mathrm{rc}}=\arcsin\left(\frac{\lambda f_{\mathrm{dc}}}{2v}\right)\\ \beta_{2}=\frac{a_{r}\sin\theta_{\mathrm{rc}}}{2}\\ \beta_{3}=-\frac{\lambda }{12}f_{2r}-\frac{a_{r}v\cos^{2}\theta_{\mathrm{rc}}}{2r_{c}}-\frac{v^{3}\cos^{2}\theta_{\mathrm{rc}}\sin\theta_{\mathrm{rc}}}{2r_{c}^{2}}\\ \beta_{4}=-\frac{\lambda }{48}f_{3r}-\frac{a_{r}^{2}\cos^{2}\theta_{\mathrm{rc}}}{8r_{c}}-\frac{3a_{r}v^{2}\cos^{2}\theta_{\mathrm{rc}}\sin\theta_{\mathrm{rc}}}{4r_{c}^{2}}-\frac{v^{4}\cos^{2}\theta_{\mathrm{rc}}\left(5\sin^{2}\theta_{\mathrm{rc}}-1\right)}{8r_{c}^{3}} \end{array}\right..$$

By comparing Equation (41) with Equation (44), it can be observed that both SEARM and MESRM account for the azimuth variation in the equivalent radar velocity. They use constant acceleration to correct the non-linear trajectory, achieving accuracy up to the fourth-order term of the actual range history. Both models consider certain higher-order terms, with differences only in their representation format.

#### 4.4.4. Advanced Equivalent Squint Range Model

The design of the range model not only considers the fitting accuracy of non-linear trajectories but also needs to facilitate the subsequent design of the imaging algorithms. As the foundation for the design of imaging algorithm, the range model determines the difficulty of decoupling range and azimuth in the subsequent process. In this paper, an advanced equivalent squint range model (AESRM) is proposed by combining the simplicity of CHRE with the accuracy of FORM.

The AESRM can be expressed as follows:

(47) $$\begin{array}{cc} R_{\mathrm{AESRM}}\left(\eta \right) & =\sqrt{r_{c}^{2}+v^{2}\eta^{2}-2r_{c}v\eta \sin\theta_{\mathrm{rc}}}+\Delta k_{3}\eta^{3}+\Delta k_{4}\eta^{4}, \end{array}$$

where $\Delta k_{3}$ and $\Delta k_{4}$ are coefficients of third- and fourth-order error terms, respectively.

By Taylor expanding Equation (47) at zero azimuth time and retaining the fourth-order term, we have:

(48) $$\begin{array}{cc} R_{\mathrm{AESRMs}}\left(\eta \right) & =r_{c}-vs.\sin\theta_{\mathrm{rc}}\eta +\frac{v^{2}\cos^{2}\theta_{\mathrm{rc}}}{2r_{c}}\eta^{2}+\left(\Delta k_{3}+\frac{v^{3}\cos^{2}\theta_{\mathrm{rc}}\sin\theta_{\mathrm{rc}}}{2r_{c}^{2}}\right)\eta^{3}\\ \; & +\left(\Delta k_{4}+\frac{v^{4}\cos^{2}\theta_{\mathrm{rc}}\left(5\sin^{2}\theta_{\mathrm{rc}}-1\right)}{8r_{c}^{3}}\right)\eta^{4}. \end{array}$$

By simultaneously solving Equations (31) and (48), we can obtain

(49) $$\left\{\begin{array}{c} v=\sqrt{{\left(\frac{\lambda f_{\mathrm{dc}}}{2}\right)}^{2}-\frac{\lambda r_{c}f_{r}}{2}}\\ \theta_{\mathrm{rc}}=\arcsin\left(\frac{\lambda f_{\mathrm{dc}}}{2v}\right)\\ \Delta k_{3}=-\frac{\lambda }{12}f_{2r}-\frac{v^{3}\cos^{2}\theta_{\mathrm{rc}}\sin\theta_{\mathrm{rc}}}{2r_{c}^{2}}\\ \Delta k_{4}=-\frac{\lambda }{48}f_{3r}-\frac{v^{4}\cos^{2}\theta_{\mathrm{rc}}\left(5\sin^{2}\theta_{\mathrm{rc}}-1\right)}{8r_{c}^{3}} \end{array}\right..$$

The proposed AESRM is based on ESRM, but with the addition of third- and fourth-order error terms, which can accurately model the fourth-order term of the actual range history and maintain the simplicity of the original hyperbolic model, demonstrating high accuracy.

To derive the two-dimensional spectrum expression of the echo signal under AESRM, we first substitute Equation (47) into Equation (1) and perform a range Fourier transform (FT) on the two-dimensional time-domain echo signal using the principle of stationary phase (POSP) [14]. Neglecting the constant term, the signal in the range frequency domain becomes

(50) $$Ss\left(f_{\tau },\eta \right)=\exp\left(-j\frac{\pi {f_{\tau }}^{2}}{K_{r}}-j\frac{4\pi \left(f_{0}+f_{\tau }\right)R_{\mathrm{AESRM}}\left(\eta \right)}{c}\right),$$

where $f_{\tau }$ represents the range frequency, and $f_{0}$ is the carrier frequency.

Next, we apply the POSP for azimuth FT to obtain the two-dimensional frequency-domain signal. The azimuth FT of Equation (50) is

(51) $$SS\left(f_{\tau },f_{\eta }\right)=\int_{-\infty }^{\infty }Ss\left(f_{\tau },\eta \right)\exp\left(-j2\pi f_{\eta }\eta \right)d\eta ,$$

where $f_{\eta }$ represents the azimuth frequency, and the phase in the Fourier integral is

(52) $$\theta \left(\eta \right)=-\frac{\pi {f_{\tau }}^{2}}{K_{r}}-\frac{4\pi \left(f_{0}+f_{\tau }\right)R_{\mathrm{AESRM}}\left(\eta \right)}{c}-2\pi f_{\eta }\eta .$$

Since the third-order and fourth-order terms in $R_{\mathrm{AESRM}}$ are relatively small, the influence of higher-order terms can be neglected when solving for the stationary points in azimuth time. By finding the solutions where θ(η) has a derivative of zero with respect to η, we can establish the relationship between azimuth time and frequency in the two-dimensional frequency domain as

(53) $$\eta_{0}=-\frac{r_{c}\cos\theta_{\mathrm{rc}}cf_{\eta }}{2v^{2}\left(f_{0}+f_{\tau }\right)\sqrt{1-\frac{c^{2}{f_{\eta }}^{2}}{4v^{2}{\left(f_{0}+f_{\tau }\right)}^{2}}}}+\frac{r_{c}\sin\theta_{\mathrm{rc}}}{v}.$$

Finally, by substituting Equation (53) into Equation (52), we can obtain the phase of the two-dimensional spectrum of the echo signal as

(54) $$\theta_{2}\mathrm{df}\left(f_{\tau },f_{\eta }\right)=-\frac{4\pi r_{c}\cos\theta_{\mathrm{rc}}}{c}\sqrt{{\left(f_{0}+f_{\tau }\right)}^{2}-\frac{c^{2}f_{\eta }^{2}}{4v^{2}}}-\frac{\pi f_{\tau }^{2}}{K_{r}}-\frac{4\pi \left(f_{0}+f_{\tau }\right)}{c}\left(\Delta k_{3}\eta_{0}^{3}+\Delta k_{4}\eta_{0}^{4}\right).$$

From the above derivation, it can be observed that the AESRM proposed in this paper provides a convenient way to obtain the two-dimensional spectrum of the echo signal. Compared to the traditional two-dimensional spectrum under a straight trajectory [14], the derived spectrum in this paper maintains its simplicity in structure, facilitating the design and improvement of subsequent imaging algorithms.

## 5. Simulation Results and Analysis

This section primarily conducts simulation and analysis of key parameters by simulating the motion of low Earth orbit (LEO) SAR. Subsequently, the effectiveness of the proposed AESRM is validated through simulation of point target in spotlight mode.

**Figure 5:**
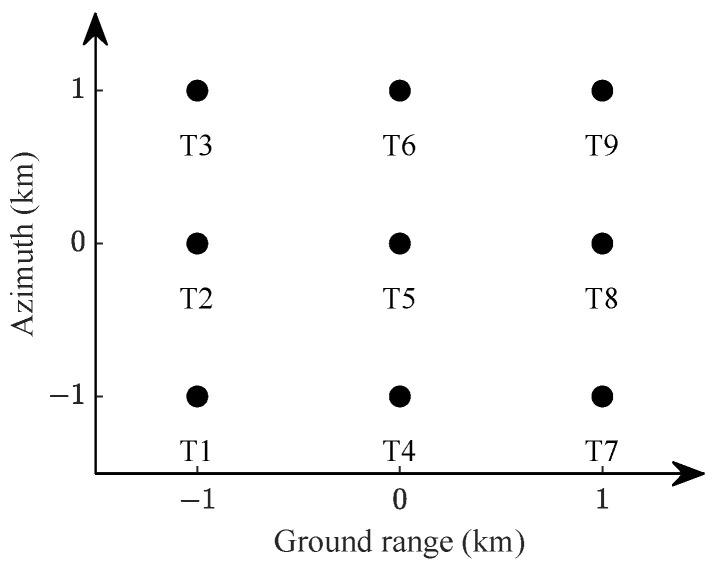
Distribution map of point targets in satellite scene coordinate system.

The point target simulation involves raw echo generation using the time-domain simulation method outlined in [32]. In this simulation, nine point targets are uniformly distributed in the satellite scene coordinate system, with the specific distribution illustrated in Figure 5. Among them, target T5 is located at the center of the scene, and the overall simulated scene size is 2km×2km. The specific input parameters for the spaceborne SAR system simulation are detailed in Table 1.

### 5.1. Satellite Parameters Simulation

**Figure 6:**
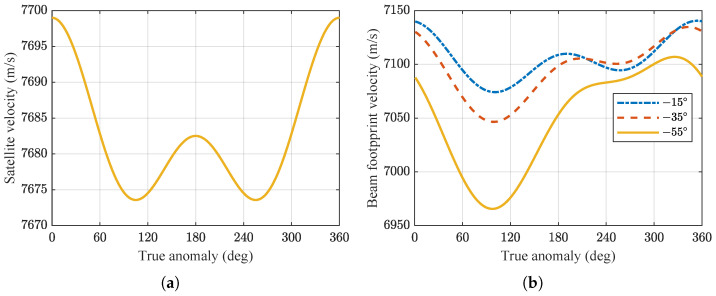
(**a**) Platform velocity $V_{s}$ of the satellite. (**b**) Antenna beam footprint velocity $V_{g}$.

For spaceborne SAR systems, the geometric relationships are more complex compared to airborne SAR systems. This complexity arises from the curvature of both the satellite orbit and the Earth’s surface, as well as the Earth’s rotation independent of the satellite orbit. In the ECR coordinate system, the platform velocity of the satellite, denoted as $V_{s}$, significantly differs from the antenna beam footprint velocity when projected onto the ground, denoted as $V_{g}$. These velocities also vary with changes in the satellite’s orbital position. Figure 6a illustrates that within one orbital cycle, $V_{s}$ is smaller at the poles and larger near the equator.

The variation of $V_{g}$ with respect to the true anomaly *f*, for different antenna looking angles, is depicted in Figure 6b. It can be observed that $V_{g}$ is much smaller compared to $V_{s}$. Additionally, they exhibit different variations at different positions along the orbit, indicating space variant in the azimuth direction.

**Figure 7:**
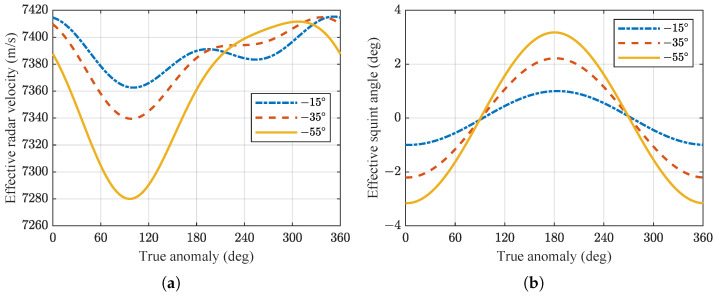
(**a**) Effective radar velocity *v*. (**b**) Effective squint angle $\theta_{\mathrm{rc}}$.

In the case of airborne SAR systems, the speed of the aircraft platform and the ground velocity of the beam coverage area are consistent. The flight path can be approximated as a straight line, allowing the range model to be described using a hyperbolic equation. However, for spaceborne SAR systems, due to the differences in spatial geometry, it is usually necessary to approximate the orbit as an equivalent straight line. This can be achieved by utilizing the effective radar velocity *v* and the effective squint angle $\theta_{\mathrm{rc}}$ at the scene center to transform the range model into a hyperbolic equation. To investigate the effects at different looking angles, simulations were conducted to examine the variations of *v* and $\theta_{\mathrm{rc}}$ with respect to the true anomaly within one orbital cycle. The simulation results are presented in Figure 7a,b.

The effective radar velocity and the effective squint angle are derived based on precise geometric modeling of spaceborne SAR, utilizing the Doppler centroid and Doppler FM rate. It can be observed that even in the case of broadside mode, the effective squint angle for spaceborne SAR is non-zero due to the curvature of the orbit and Earth’s rotation. It is maximum near the equator and minimum at the poles, as illustrated in Figure 7b.

**Figure 8:**
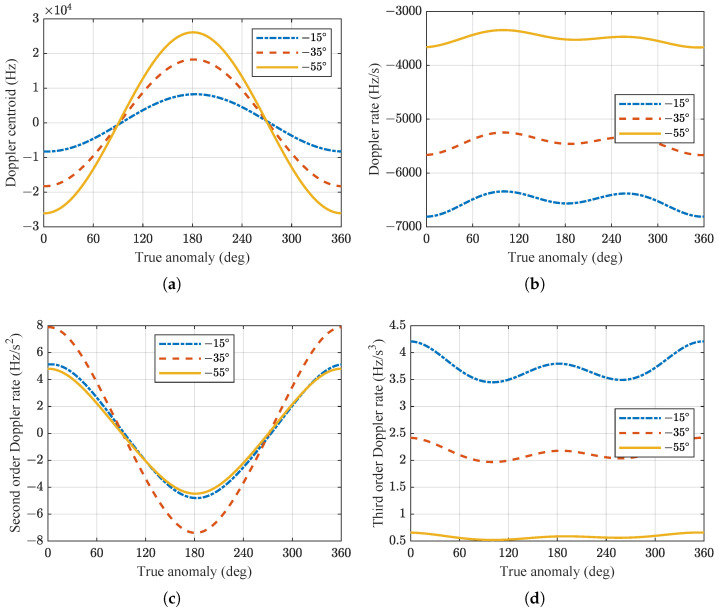
(**a**–**d**) Correspond to the variations in the 1st- to 4th-order Doppler parameters with respect to the true anomaly at different looking angles.

Based on the simulation parameters of the spaceborne SAR system given in Table 1, simulations were conducted for the 1st–4th order Doppler parameters at different positions of the spaceborne SAR orbit for various looking angles, as shown in Figure 8. The simulations did not consider perturbations from other celestial bodies and did not apply attitude steering to the satellite.

Due to the influence of the eccentricity of the orbit and the rotation of the Earth, the Doppler parameters have different values at different positions along the orbit. Figure 8a illustrates that even when the radar operates in the broadside mode, the Doppler centroid is not zero and is greatly affected by the Earth’s rotation. The Doppler centroid corresponds to the highest relative velocity of targets near the equator, whereas it approaches zero near the Earth’s poles.

### 5.2. Simulation of Range Model Errors

Through precise satellite–Earth geometric modeling, under the assumptions of a satellite two-body orbit model and the Earth’s WGS84 model, accurate position vectors of the satellite and targets are obtained using space coordinate transformation and vector analysis. The resulting ACNM has high accuracy and can serve as a criterion for evaluating the fitting accuracy of various range models [34]. The azimuth phase history errors caused by range model errors [21] can be expressed as:

(55) $$\Delta \varphi =-\frac{4\pi \left(R_{\mathrm{model}}\left(\eta \right)-R_{\mathrm{ACNM}}\left(\eta \right)\right)}{\lambda }$$

where $R_{\mathrm{model}}\left(\eta \right)$ represents the fitted value of range models, and different range models have varying accuracy in fitting range history under nonlinear trajectories.

**Figure 9:**
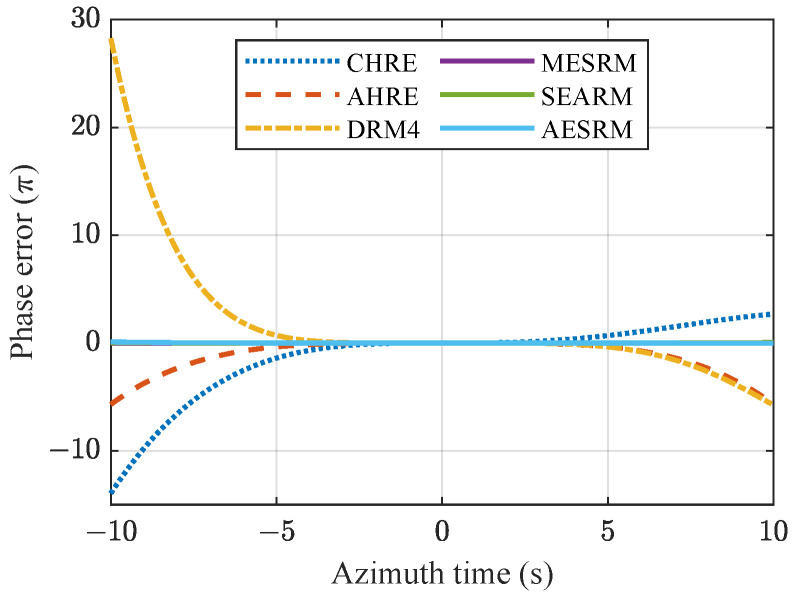
Phase error as a function of azimuth time for different range models.

Based on the simulation parameters in Table 1, simulations were conducted for the range models described in Section 4. Figure 9 illustrates the variation in phase errors generated by different range models with respect to azimuth time. When the absolute value of the computed phase error exceeds π/4, it indicates that the corresponding range model will introduce azimuth defocusing in SAR data focusing processing [37].

From Figure 9, it can be observed that with an increase in azimuth time, the errors generated by the range models gradually increase. Due to the different order fittings of each range model to the true range history, the errors produced are also different. Among them, CHRE has the lowest accuracy because it only accurately fits the true range history up to the first two terms of the Taylor expansion, whereas AHRE improves the accuracy by compensating for the linear part of the higher-order error terms based on CHRE. FORM considers the four terms, further improving the accuracy. The three curves of MESRM (purple line), SEARM (green line), and the proposed AESRM (blue line) are almost overlapping, indicating similar accuracies. All three models are built upon CHRE and can accurately fit up to the fourth-order term while considering some higher-order terms. This enables them to achieve higher accuracy compared to other models.

**Figure 10:**
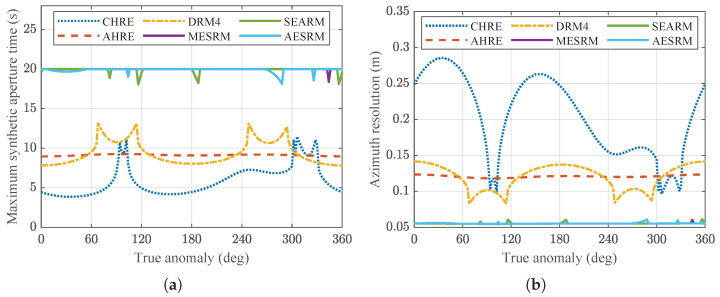
Variation in performance for different range models with respect to the true anomaly. (**a**) Maximum synthetic aperture time and (**b**) azimuth resolution.

As the azimuth time increases, the errors generated by the range models also increase. Therefore, the accuracy of a model can be measured by the length of the maximum synthetic aperture time that satisfies a phase error less than π/4. Since the relative velocity of the spaceborne SAR with respect to the ground target varies at different positions along the orbit, and the curvature of the orbit is also different, the maximum synthetic aperture time that each range model can fit is constantly changing. According to the parameters in Table 1, Figure 10a simulates the maximum synthetic aperture time for different range models at different positions along the orbit. The azimuth duration set in the simulation is 20 s.

Due to the influence of Earth rotation and satellite elliptical orbit, the maximum synthetic aperture time for different range models at different positions along the orbit is different. From Figure 10a, it can be seen that the maximum synthetic aperture time for all range models varies with the position along the satellite orbit. The effective maximum synthetic aperture time for CHRE, AHRE, DRM4, MESRM, SEARM, and AESRM are 3.86 s, 8.97 s, 7.82 s, 18.39 s, 18.05 s, and 18.14 s, respectively. Since the Doppler parameters change with the orbit position, the accuracy of the range model also varies. When the satellite is above the Earth’s poles, i.e., when the true anomaly *f* is equal to $90^{\circ }$ and $270^{\circ }$, the errors are small and the achievable synthetic aperture time limit is relatively large because the higher-order Doppler components, especially the third-order term, are close to zero. However, near the equator, the higher-order components, especially the third-order component, are relatively large. If the range model does not consider the third-order term, the approximate errors introduced by the slant range will be significant. The asymmetry of the curves near the poles and the equator in the figure is due to the inconsistent side-viewing direction of the radar antenna beam and the Earth rotation direction during the forward and backward parts of the orbit.

Figure 10b presents the highest-achievable azimuth resolution for different range models at different positions along the orbit. Since the third-order Doppler parameters change significantly throughout the orbit, and CHRE only includes up to the second-order term, the error of CHRE changes significantly with the orbit position. The other range models are relatively stable and achieve relatively high resolution. In SAR imaging algorithm design, according to the resolution requirements, it is generally necessary to ensure that the phase error introduced by the range model throughout the orbit does not exceed π/4, otherwise, the target cannot be well focused.

MESRM, SEARM, and proposed AESRM achieve a maximum synthetic aperture time at certain positions along the orbit that exceeds 20 s. However, to ensure the best imaging capability throughout the entire orbit, evaluating the accuracy of a range model requires considering its minimum synthetic aperture time throughout the entire orbit. It should be noted that the above comparative results are based on the system parameters in Table 1 obtained from simulation analysis. The optimal performance of various range models may be affected by the parameters, and here we only provide a general analytical method.

### 5.3. Point Target Simulation

To compare the improvement of AESRM proposed in this paper relative to ESRM, we adopted the enhanced extended ωK algorithm, which incorporates second-order azimuth compression to address the range-variant effect of equivalent radar velocity based on the original algorithm [16,38].

To process high-resolution spaceborne SAR data, traditional focusing algorithms encounter several limitations, such as orbit curvature, stop-and-go approximation, azimuth spectrum aliasing, and precise imaging algorithms [12,39].

**Figure 11:**
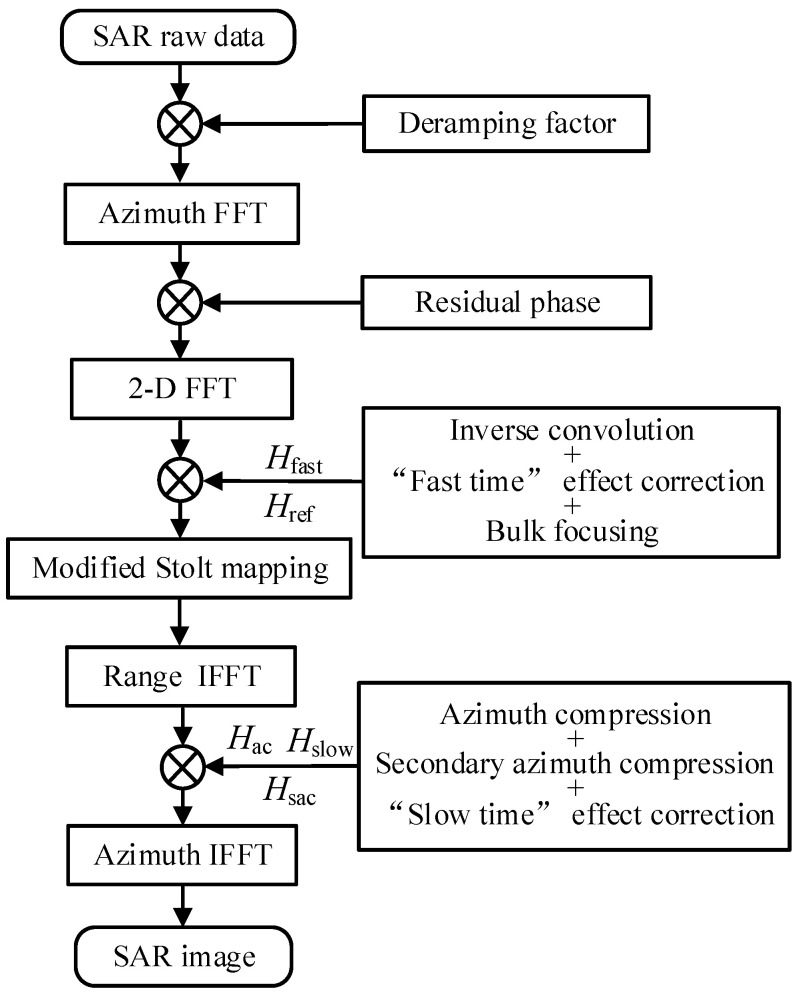
Flowchart of the proposed algorithm.

To overcome these limitations, we adopt AESRM to fit the nonlinear trajectory under orbit curvature. To mitigate errors introduced by the stop-and-go approximation in high-resolution scenarios, we employ fast-time and slow-time effects corrections [9]. When the azimuth resolution is high, leading to an azimuth bandwidth exceeding the actual pulse eepetition frequency, we apply a two-step processing approach [39] to address the azimuth spectrum aliasing issue. Finally, we use our proposed modified EOKA to precisely focus the post-processed data, which are free from azimuth ambiguities. An illustration of the improved imaging processing workflow is presented in Figure 11.

**Figure 12:**
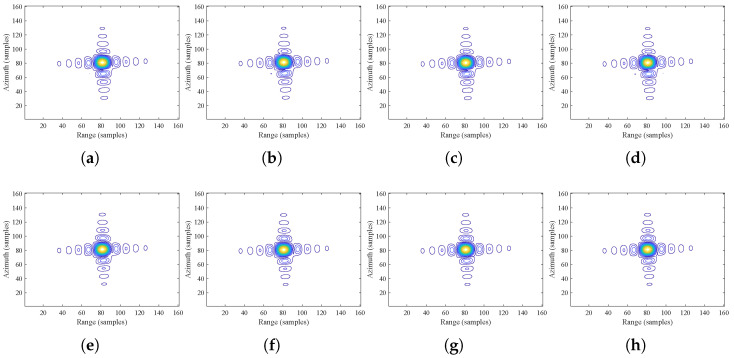
Contour maps of the target after processing based on different range models. (**a**–**d**) traditional ESRM, (**e**–**h**) proposed AESRM in this paper. The first to fourth columns correspond to targets T5, T6, T8, and T9, respectively.

Figure 12 presents the contour maps of point target imaging results for the selected typical targets T5, T6, T8, and T9 under two range models, where the top row corresponds to the imaging results of ESRM, and the bottom row corresponds to the imaging results of AESRM considering the non-linear trajectory.

From Figure 12, it is observed that ESRM, by neglecting terms higher than the third-order in actual range history, results in asymmetric sidelobes in the azimuth direction. In contrast, the proposed AESRM accurately fits up to the fourth order of actual range history and compensates for some higher-order terms, leading to more symmetrical sidelobes in the imaging results.

**Figure 13:**
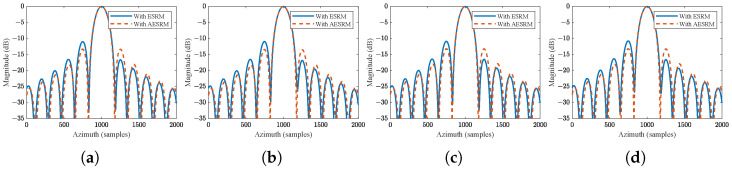
Comparison of azimuth profiles of the focusing results using ESRM and proposed AESRM. (**a**–**d**) correspond to targets T5, T6, T8, and T9, respectively.

Furthermore, Figure 13 further illustrates the azimuth profile comparison of imaging results under the two range models. It is evident that the proposed AESRM, compared to the traditional ESRM, effectively addresses the asymmetry issue in the azimuth sidelobes caused by fitting errors of the range history due to the nonlinear characteristics of the motion trajectory in high-resolution spaceborne SAR.

To further quantitatively evaluate and compare the imaging results, we measured the quality parameters of the targets. Table 2 presents the comparative results of impulse response width (IRW), peak sidelobe ratio (PSLR), and integrated sidelobe ratio (ISLR) under the two range models. No weighting was applied during the imaging process, and the ideal focusing effect should resemble a two-dimensional sinc function.

In high-resolution scenarios, the motion trajectory in spaceborne SAR exhibits significant nonlinear characteristics. The comparative results in Table 2 reveal that traditional ESRM fails to accurately fit the actual range history, resulting in errors that mainly affect the azimuthal focusing, leading to asymmetric azimuth sidelobes and an increased PSLR. By using the proposed AESRM, a better fit to the actual range history is achieved, and all measured parameters approach the ideal values, enabling improved target focusing.

## 6. Conclusions

In this study, we proposed an AESRM to address the challenges of developing an accurate range model for high-resolution spaceborne SAR. By analyzing the motion and Doppler parameters of the satellite and ground targets, the AESRM accurately fits the actual range history while maintaining the simplicity of the traditional model and introducing third-order and fourth-order error terms. Through experimental simulations, we have verified the effectiveness of the proposed AESRM. The results demonstrate that the AESRM improves the fitting accuracy of the range history under nonlinear trajectories, providing a more precise representation of the actual range history and enhancing the focusing effect in high-resolution spaceborne SAR imaging. This advancement lays a solid foundation for the design of imaging algorithms, enabling higher resolution and more accurate radar imaging. Future research could explore the development of high-efficiency and high-precision imaging algorithms based on AESRM.

## Figures and Tables

**Table 1:** Simulation parameters for the spaceborne SAR experiment.

Parameters	Value	Unit
Semimajor axis	6883.513	km
Inclination	97.44	deg
Eccentricity	0.0011	-
Longitude ascending node	0	deg
Argument of perigee	0	deg
Center time location	$5T_{s}/8$	s
Earth Model	WGS-84	-
Antenna’s azimuth diameter	4.2	m
Antenna’s elevation diameter	2	m
Looking angle (right-looking)	−15/−35/−55	deg
Azimuth angle	0	deg
Carrier frequency	9.6	GHz
Pulse duration	40	μs
Chirp bandwidth	500	MHz
Sampling frequency	600	MHz
Pulse repetition frequency	3500	Hz
Synthetic aperture time	6	s

**Table 2:** Comparison of point target quality parameters for the focusing results using ESRM and proposed AESRM.

Target	Range			Azimuth		
	PSLR (dB)	ISLR (dB)	IRW(dm)	PSLR (dB)	ISLR (dB)	IRW(dm)
Ideal	−13.26	−9.94	2.67	−13.26	−9.94	2.03
T5	−13.44/−13.43	−10.18/−10.19	2.67/2.66	−10.97/−13.31	−9.87/−10.31	1.96/ 1.96
T6	−13.44/−13.43	−10.19/−10.2	2.67/2.67	−10.94/−13.42	−9.91/−10.45	1.97/1.96
T8	−13.43/−13.43	−10.19/−10.17	2.66/2.66	−10.87/−13.18	−9.74/−10.13	1.96/1.95
T9	−13.44/−13.43	−10.18/−10.18	2.66/2.66	−10.77/−13.20	−9.66/−10.15	1.96/ 1.95

## Data Availability

Data are contained within the article.

## Abbreviations

The following symbols and abbreviations are used in this manuscript:

*a*	Semi-major axis (meters)
$A_{\left(\cdot \cdot \right)}$	Coordinate transformation matrix
$a_{\left(\cdot \right)}$	Acceleration vector (meters/second²)
*c*	Speed of light (meters/second)
*e*	Orbital eccentricity
*E*	Eccentric anomaly
$E_{\left(\cdot \right)}$	Coordinate system
*f*	True anomaly
$f_{0}$	Carrier frequency (hertz)
$f_{r}$	Doppler frequency (hertz)
$f_{\eta }$	Azimuth (Doppler) frequency (hertz)
$f_{\tau }$	Range frequency (hertz)
$f_{\mathrm{dc}}$	Absolute Doppler center frequency (hertz)
*i*	Orbital inclination (degrees)
$K_{r}$	Frequency modulation rate of transmitted pulse chirp (hertz/second)
*M*	Mean anomaly
$r_{\left(\cdot \right)}$	Position vector (meters)
$r_{0}$	Slant range of closest approach (meters)
$r_{c}$	Instantaneous slant range to synthetic aperture center (meters)
$r_{\mathrm{ref}}$	Reference range (meters)
$R_{e}$	Earth’s semi-major axis (meters)
$R_{p}$	Earth’s semi-minor axis (meters)
R(η)	Time-domain slant range (meters)
$R_{s}$	Satellite polar radius (meters)
$R_{\mathrm{ta}}$	Earth radius at target latitude circle (meters)
$R_{\left(\cdot \right)}$	Rotation matrix
$T_{a}$	Azimuth exposure time (seconds)
$T_{r}$	Transmitted pulse time duration (seconds)
*v*	Equivalent radar velocity (meters/second)
$v_{\mathrm{ref}}$	Equivalent radar velocity at reference range (meters/second)
$v_{\left(\cdot \right)}$	Velocity vector (meters/second)
$V_{g}$	Antenna beam footprint velocity when projected onto theground, a scalar and always
	positive (meters/second)
$V_{s}$	Velocity of radar platform along flight trajectory (meters/second)
η	Azimuth time (seconds)
$\eta_{c}$	Beam center crossing time (seconds)
$\theta_{\mathrm{lat}}$	Target latitude (degrees)
$\theta_{\mathrm{lon}}$	Target longitude (degrees)
$\theta_{r}$	General squint angle in rectilinear geometry, measured from zero Doppler (degrees)
$\theta_{rc}$	Squint angle of the radar beam center in rectilinear geometry, measured from
	zero Doppler (degrees)
λ	Wavelength of carrier frequency (meters)
τ	Range time (seconds)
Ω	Right ascension of the ascending node (degrees)
ω	Argument of periapsis (degrees)
$\omega_{e}$	Angular velocity of Earth’s rotation (radians/second)
μ	Gravitational constant (meters³/second²)
ACNM	accurate coordinate numerical model
AESRM	advanced equivalent squint range model
AHRE	advanced hyperbolic range equation
CHRE	conventional hyperbolic range equation
ECI	Earth-centered inertial (coordinate system)
ECR	Earth-centered rotating (coordinate system)
ESRM	equivalent squint range model
FT	Fourier transform
FM	frequency modulation
FORM	fourth-order range model
IRW	impulse response width (resolution)
ISLR	integrated sidelobe ratio
LEO	low Earth orbit
LFM	linear frequency modulation
MCRM	motion compensation range model
MESRM	modified equivalent squint range model
MSR	method of series reversion
PRF	pulse repetition frequency
POSP	principle of stationary phase
PSLR	peak sidelobe ratio (ratio to main lobe)
RAAN	right ascension of the ascending node
SAR	synthetic aperture radar
SEARM	squint equivalent acceleration range model
WGS-84	World Geodetic System defined in 1984
